# Anti-Typhoid Vaccine

**Published:** 1908-02-22

**Authors:** 


### Points in Treatment.

### ANTI-TYPHOID VACCINE.

Though chiefly used in the Army, particularly in connection with troops going abroad, the use of a vaccine as a prophylactic measure against infection with typhoid fever is sometimes required in ordinary practice. So much has been written upon the subject in the lay Press that the public know of the possibility of this form of treatment, and people who propose going to foreign parts, such as Ceylon, South Africa, and elsewhere where typhoid fever may be contracted far from skilled medical attention, not infrequently ask their doctors whether they should have the prophylactic before going there. Sometimes, indeed, they almost insist upon having it. In any case it is important to be able to tell such a patient exactly what the treatment is, and what are its pros and cons.

There are two very different things which are sometimes apt to be confused with one another in this connection, and these are the anti-typhoid *serum* and the anti-typhoid *vaccine*. The *serum* has been advocated in the treatment of existing typhoid fever, upon the same lines, theoretically, as the treatment of existing diphtheria with anti-diphtheritic serum. It is not with this anti-typhoid serum that we are at present dealing, but it may be remarked incidentally that there is practically no evidence that such serum treatment is of the least use as a curative measure in typhoid fever. It is possible that a serum for the cure of typhoid fever may some day be discovered, but that which is at present available is to all intents and purposes useless; indeed, it is worse than useless, for not only does it fail to cure, but also it renders the patient liable to the urticarial eruptions and other phenomena of "serum disease" in addition to the unaltered symptoms of typhoid fever. Moreover, the serum, given to a healthy person, seems to have no influence as a prophylactic. The anti-typhoid *vaccine*, on the other hand, is prophylactic, and it has many advocates. The general conclusions drawn by those who have had an opportunity of watching its effects in many cases are briefly these:

The actual inoculations are moderately painful and are accompanied by immediate and transitory constitutional symptoms. For six weeks or so after the inoculations the patient has an *increased* susceptibility to typhoid fever; thereafter, his liability to contract the disease is very considerably diminished, and, moreover, even if the fever is caught, its course is more benign, and the prognosis considerably better, than is the case in a non-inoculated person under the same circumstances. Conversation with soldiers who went through the South African War shows that they have a firm belief in two things: first, that they very much dislike the actual effects of the inoculations; secondly, that notwithstanding this dislike they are fully assured that they would submit to the inoculation again, and would advise others under the circumstances to do likewise, because they feel sure that without them they run a much greater risk of dying of enteric fever when they catch the disease.

The usual practice in the case of the troops was to inoculate them on board ship. This, however, has two great disadvantages which should be avoided in the case of private patients, and indeed in all cases. These are: first, that the combined effects of the inoculations and of sea-sickness are sometimes pitiable; and, secondly, that the patient arrives at the end of the journey within the six weeks, and therefore at a time when susceptibility to typhoid fever is greater instead of less. If, therefore, anti-typhoid innoculation is to be performed, it is best done before the patient sails; and it is best performed in two stages, using a small dose of the vaccine first, and then a bigger dose ten days later.

The vaccine is prepared, like other vaccines, by growing the typhoid bacilli in a fluid medium, and then sterilising the culture. The products of the bacteria thus obtained are measured off into small glass bulbs, hermetically sealed and sterile, ready for injection. As a rule, the first dose contains the products of 500,000,000 bacilli, and the second the products of 1,000,000,000. The volume of fluid is quite small in either case, a little over five drops (0.5 c.c.) in the first instance, and a little over ten drops (1 c.c.) in the second. The advantage of having the volume of the dose as small as this is that, on breaking the neck of the sealed glass bulb, the contents are easily taken up in an ordinary hypodermic syringe, rendered aseptic, and the injection can be given beneath the skin of the arm, or any part of the body, just as an injection of strychnine or of morphia is given.

After the first and smaller dose the patient should immediately rest, preferably in bed, for in nearly every case both local and constitutional symptoms come on soon after the injection, and they are considerably worse if the patient is not lying down. The local symptoms are redness, swelling, pain, and tenderness at the site of inoculation. The constitutional symptoms are malaise, loss of appetite, a feeling of nausea, dull headache, and slight pyrexia. Broadly speaking, the more severe the local symptoms are the less marked is the constitutional reaction, and *vice versa*; and both disappear usually within twelve hours, and nearly always within thirty-six hours.

After the second and larger dose, administered ten days later, the local and general effects are very much less obvious than they are after the first; indeed, there may be no trouble at all upon the second occasion, though the same precautions should be adopted as after the first. In the earlier cases, the inoculations were made in one large dose, and the effects were so severe that the method of using two smaller doses at an interval of ten days was devised; the results are now very much better.

The cause of the local reaction at the site of injection is probably, in part at least, the diminution in the coagulability of the blood produced by the vaccine; at any rate, by giving calcium lactate in five grain doses, at intervals of four hours for six doses, the trouble there is often considerably mitigated.

